# The probability of diabetes and hypertension by levels of neighborhood walkability and traffic-related air pollution across 15 municipalities in Southern Ontario, Canada: A dataset derived from 2,496,458 community dwelling-adults

**DOI:** 10.1016/j.dib.2019.104439

**Published:** 2019-08-28

**Authors:** Nicholas A. Howell, Jack V. Tu, Rahim Moineddin, Hong Chen, Anna Chu, Perry Hystad, Gillian L. Booth

**Affiliations:** a Centre for Urban Health Solutions, Li Ka Shing Knowledge Institute, St. Michael's Hospital, 209 Victoria Street, Ontario, Ontario, M5B 1T8, Canada; b Institute for Health Policy, Management, and Evaluation, Dalla Lana School of Public Health, University of Toronto, 155 College Street, Toronto, Ontario, M5T 3M6, Canada; c Institute for Clinical and Evaluative Sciences, 2075 Bayview Avenue, Toronto, Ontario, M4N 3M5, Canada; d Schulich Heart Centre, Sunnybrook Health Sciences Centre, 2075 Bayview Avenue, Toronto, Ontario, M4N 3M5, Canada; e Department of Medicine, University of Toronto, 190 Elizabeth Street, Toronto, Ontario, M5G 2C4, Canada; f Department of Family and Community Medicine, Faculty of Medicine, University of Toronto, 500 University Avenue, Toronto, Ontario, M5G 1V7, Canada; g Public Health Ontario, 480 University Ave, Toronto, Ontario, M5G 1V2, Canada; h Dalla Lana School of Public Health, University of Toronto, 155 College Street, Toronto, Ontario, M5T 3M7, Canada; i College of Public Health and Human Sciences, Oregon State University, 160 SW 26th St., Corvallis, OR, 97331, United States of America

**Keywords:** Walkability, Traffic-related air pollution, NO_2_, Diabetes, Hypertension, Cardiovascular risk factors, Health administrative data

## Abstract

Individuals’ risk for cardiovascular disease is shaped by lifestyle factors such as participation in physical activity. Some studies have suggested that rates of physical activity may be higher in walkable neighborhoods that are more supportive of engaging in physical activity in daily life. However, walkable neighborhoods may also contain increased levels of traffic-related air pollution (TRAP). Traffic-related air pollution, often measured through a surrogate marker (e.g. NO_2_), has been associated cardiovascular disease risk and risk factors [1], [2], [3], [4]. The higher levels of TRAP in walkable neighborhoods may in turn increase the likelihood of developing conditions like hypertension and diabetes. Our recent work assessed how walkability and TRAP jointly affect the odds of diabetes and hypertension in a sample of community-dwelling adults from Southern Ontario, Canada [5]. This article contains additional data on the probability and odds of hypertension and diabetes according to their walkability and TRAP exposures. Data on cardiovascular risk factors were collected using health administrative databases and environmental exposures were assessed using national land use regression models predicting ground level concentrations of NO_2_ and validated walkability indices. The included data were generated using logistic regression accounting for exposures, covariates, and neighborhood clustering. These data may be used as primary data in future health risk assessments and systematic reviews, or to aid in the design of studies examining interactions between built environment and TRAP exposures (e.g. sample size calculations).

**Table:** Specifications Table

Subject	Public Health and Health Policy
Specific subject area	Environmental Epidemiology
Type of data	Table
How data were acquired	Administrative health care data of residents receiving coverage under the Ontario Health Insurance Plan, offered to all permanent residents in Ontario, Canada. Model based estimates of probability of hypertension and diabetes using logistic regression. Models estimated using SAS Version 9.4 (SAS Institute, Cary, NC).
Data format	Analyzed
Parameters for data collection	Data were collected from population-based samples of community-dwelling individuals in Southern Ontario, Canada. Traffic-related air pollution exposures were assessed using a national model of ground-level NO_2_ concentration.
Description of data collection	Clinical and socio-demographic data were collected using health administrative databases. Walkability exposures were collected from a validated database of neighborhood-level built environment characteristics across 16 municipalities. NO_2_ exposure information was collected from a national pollution model predicting ground-level concentration at postal codes across Canada.
Data source location	City/Town/Region: 16 Municipalities in Southern Ontario (including Toronto & the Greater Toronto Area, Hamilton, London, and Ottawa) Country: Canada
Data accessibility	Analyzed data included with the article. The data set from this study is held securely in coded form at ICES [6]. While data sharing agreements prohibit ICES from making the data set publicly available, access may be granted to those who meet pre-specified criteria for confidential access, available at www.ices.on.ca/DAS. The full data set creation plan and underlying analytic code are available from the authors upon request, understanding that the programs may rely upon coding templates or macros that are unique to ICES.
Related research article	Nicholas A. Howell, Jack V Tu, Rahim Moineddin, Hong Chen, Anna Chu, Perry Hystad, Gillian L. Booth Interaction between neighborhood walkability and traffic-related air pollution on hypertension and diabetes: The CANHEART cohort Environment International https://doi.org/10.1016/j.envint.2019.04.070

**Table:** 

**Value of the data** • Previous work examining relationships between the built environment, traffic-related air pollution, and cardiovascular risk factors has generally treated these variables in isolation. These results demonstrate how antagonistic interactions between walkability or traffic-related air pollution and cardiovascular risk factors may occur • Researchers investigating healthy community design, public health practitioners, and individuals engaged in urban policy may benefit from these data • The results reported here may be used to develop health risk assessments which take into account interactions between environmental variables, in systematic reviews of environmental correlates of cardiovascular disease risk factors, and in planning future studies examining interactions between built environment and air pollution variables • Previous analyses (e.g. Refs. [7], [8]) have used literature-derived estimates of associations between physical activity, air pollution, and cardiovascular health to assess whether the protective value of physical activity declines in polluted environments. These estimates, however, often do not consider interactions between these pollution and walkable environments. These data may provide more accurate assessments of the value of walkable environments in the context of air pollution. They may also help in the design of policies directed at mitigating air pollution in urban environments.

## 1 Data

The raw data used here are held by ICES [6]. These data were derived from a cross-sectional study of 2,496,458 adults aged 40–74 years who were living in one of 16 urban municipalities in Southern Ontario on January 1, 2008. All individuals were eligible for provincial health insurance for at least two years at inclusion, had not resided in a long-term care facility within the previous 5 years, and were free from cardiovascular disease at baseline (i.e. history of prior myocardial infarction, stroke, congestive heart failure or cardiovascular revascularization procedure). Associations between neighborhood walkability, traffic-related air pollution, hypertension and diabetes are displayed in Table 1. All estimates were adjusted for baseline sociodemographic variables, chronic obstructive pulmonary disease, the total number of individual comorbidities, and city/region. Adjusted probabilities from models including an interaction between walkability and traffic-related air pollution were estimated across levels of NO_2_ (0 ppb, 5 ppb, 10 ppb, 20 ppb, 30 ppb, 40 ppb; range of NO_2_ in sample: 3.94 ppb–51.47 ppb) and walkability (quintiles – Q1 lowest 20%, Q5 highest 20%; range in underlying walkability scores (unitless): 6.26, 27.78) (Table 2, Table 3).

**Table 1:** Associations of walkability and traffic-related air pollution with hypertension and diabetes adjusted for baseline sociodemographic factors, COPD, number of comorbidities, and city/region.

Variable	Hypertension		Diabetes	
	Independent Models	Joint Models	Independent Models	Joint Models
	OR (95% CI)	OR (95% CI)	OR (95% CI)	OR (95% CI)
Walkability Quintile				
Q1 (low)	1.24 (1.21, 1.26)	1.24 (1.22, 1.26)	1.15 (1.11, 1.18)	1.17 (1.14, 1.21)
Q2	1.24 (1.22, 1.26)	1.24 (1.22, 1.26)	1.14 (1.11, 1.17)	1.16 (1.13, 1.19)
Q3	1.21 (1.19, 1.23)	1.21 (1.19, 1.24)	1.13 (1.10, 1.15)	1.14 (1.11, 1.17)
Q4	1.16 (1.14, 1.18)	1.16 (1.14, 1.18)	1.12 (1.09, 1.15)	1.13 (1.10, 1.16)
Q5 (high)	Ref	Ref	Ref	Ref
*p* for trend	<0.0001	<0.0001	<0.0001	<0.0001
Traffic-related air pollution				
NO_2_	0.98 (0.97, 0.99)	1.00 (0.99, 1.02)	1.09 (1.07, 1.11)	1.11 (1.09, 1.13)

*Notes* Covariates included in model: age, sex, ethnicity, immigration history, neighborhood median income, COPD, number of comorbidities, and region). Association estimates for traffic-related air pollution are per 10-unit increase in NO_2_. Independent models include either walkability or traffic-related air pollution. Joint models include walkability and traffic-related air pollution simultaneously. OR: odds ratio, CI: confidence interval, Ref: reference category.

**Table 2:** Predicted probability of hypertension at varying levels of walkability and NO_2_ adjusted for baseline sociodemographic factors, COPD, number of comorbidities, and city/region.

Walkability Quintiles (Q)	NO_2_ 0 ppb (SEM)	NO_2_ 5 ppb (SEM)	NO_2_ 10 ppb (SEM)	NO_2_ 20 ppb (SEM)	NO_2_ 30 ppb (SEM)	NO_2_ 40 ppb (SEM)
Q1 (lowest)	0.22 (0.003)	0.22 (0.002)	0.22 (0.002)	0.21 (0.002)	0.21 (0.003)	0.20 (0.005)
Q2	0.22 (0.003)	0.22 (0.002)	0.22 (0.002)	0.21 (0.002)	0.21 (0.003)	0.21 (0.005)
Q3	0.21 (0.003)	0.21 (0.002)	0.21 (0.002)	0.21 (0.001)	0.21 (0.003)	0.21 (0.004)
Q4	0.20 (0.004)	0.20 (0.003)	0.20 (0.002)	0.20 (0.001)	0.21 (0.003)	0.21 (0.005)
Q5 (Highest)	0.15 (0.005)	0.16 (0.004)	0.16 (0.003)	0.18 (0.001)	0.19 (0.003)	0.21 (0.006)

*Notes:* Covariates included in model: age, sex, ethnicity, immigration history, neighborhood median income, COPD, number of comorbidities, and region. All covariates fixed at weighted average of levels for categorical covariates or mean value for continuous covariates. ppb: parts per billion. SEM: standard error of the mean.

**Table 3:** Predicted probability of diabetes at varying levels of walkability and NO_2_ adjusted for baseline sociodemographic factors, COPD, number of comorbidities, and city/region.

Walkability Quintiles (Q)	NO_2_ 0 ppb (SEM)	NO_2_ 5 ppb (SEM)	NO_2_ 10 ppb (SEM)	NO_2_ 20 ppb (SEM)	NO_2_ 30 ppb (SEM)	NO_2_ 40 ppb (SEM)
Q1 (lowest)	0.09 (0.002)	0.09 (0.002)	0.10 (0.001)	0.11 (0.001)	0.12 (0.003)	0.13 (0.005)
Q2	0.09 (0.003)	0.09 (0.002)	0.10 (0.001)	0.10 (0.001)	0.11 (0.003)	0.12 (0.005)
Q3	0.09 (0.002)	0.09 (0.002)	0.10 (0.001)	0.10 (0.001)	0.11 (0.002)	0.12 (0.004)
Q4	0.09 (0.003)	0.09 (0.002)	0.09 (0.002)	0.10 (0.001)	0.11 (0.002)	0.12 (0.004)
Q5 (Highest)	0.06 (0.003)	0.07 (0.002)	0.07 (0.002)	0.09 (0.001)	0.11 (0.003)	0.14 (0.006)

*Notes:* Covariates included in model: age, sex, ethnicity, immigration history, neighborhood median income, COPD, number of comorbidities, and region. All covariates fixed at weighted average of levels for categorical covariates or mean value for continuous covariates. ppb: parts per billion. SEM: standard error of the mean.

Associations observed among members of this same cohort who had a longer duration of exposure (resided in their residential neighborhood for at least 5 years, N = 1,609,247) are shown in Table 4. Estimated probabilities from this sample by level of walkability and traffic-related air pollution are shown in Table 5, Table 6.

**Table 4:** Associations of walkability and traffic-related air pollution with hypertension and diabetes among individuals remaining in their neighborhood for 5 or more years adjusted for baseline sociodemographic factors.

Variable	Hypertension		Diabetes	
	Independent Models	Joint Models	Independent Models	Joint Models
	OR (95% CI)	OR (95% CI)	OR (95% CI)	OR (95% CI)
Walkability Quintile				
Q1 (low)	1.29 (1.26, 1.32)	1.35 (1.32, 1.38)	1.16 (1.13, 1.19)	1.25 (1.22, 1.29)
Q2	1.28 (1.26, 1.31)	1.33 (1.30, 1.36)	1.14 (1.11, 1.17)	1.21 (1.18, 1.24)
Q3	1.26 (1.23, 1.28)	1.29 (1.27, 1.32)	1.14 (1.11, 1.17)	1.19 (1.16, 1.22)
Q4	1.17 (1.14, 1.19)	1.19 (1.17, 1.21)	1.11 (1.08, 1.14)	1.15 (1.12, 1.18)
Q5 (high)	Ref	Ref	Ref	Ref
*p* for trend	<0.0001	<0.0001	<0.0001	<0.0001
Traffic-related air pollution				
NO_2_	1.01 (1.00, 1.02)	1.08 (1.07, 1.09)	1.10 (1.09, 1.12)	1.15 (1.13, 1.17)

*Notes* Covariates included in model: age, sex, ethnicity, immigration history, neighborhood median income). Association estimates for traffic-related air pollution are per 10-unit increase in NO_2_. Independent models include either walkability or traffic-related air pollution. Joint models include walkability and traffic-related air pollution simultaneously. OR: odds ratio, CI: confidence interval, Ref: reference category.

**Table 5:** Predicted probability of hypertension at varying levels of walkability and NO_2_ among individuals remaining in their neighborhood for 5 or more years adjusted for baseline sociodemographic factors.

Walkability Quintiles (Q)	NO_2_ 0 ppb (SEM)	NO_2_ 5 ppb (SEM)	NO_2_ 10 ppb (SEM)	NO_2_ 20 ppb (SEM)	NO_2_ 30 ppb (SEM)	NO_2_ 40 ppb (SEM)
Q1 (lowest)	0.20 (0.003)	0.21 (0.003)	0.21 (0.002)	0.22 (0.002)	0.23 (0.004)	0.24 (0.006)
Q2	0.21 (0.004)	0.21 (0.003)	0.21 (0.002)	0.22 (0.002)	0.22 (0.003)	0.23 (0.005)
Q3	0.20 (0.003)	0.20 (0.003)	0.21 (0.002)	0.21 (0.002)	0.22 (0.003)	0.23 (0.005)
Q4	0.17 (0.004)	0.18 (0.003)	0.18 (0.002)	0.20 (0.002)	0.22 (0.003)	0.24 (0.005)
Q5 (Highest)	0.11 (0.005)	0.12 (0.004)	0.14 (0.003)	0.17 (0.002)	0.21 (0.004)	0.26 (0.008)

*Notes:* Covariates included in model: age, sex, ethnicity, immigration history, neighborhood median income. All covariates fixed at weighted average of levels for categorical covariates or mean value for continuous covariates. ppb: parts per billion. SEM: standard error of the mean.

**Table 6:** Predicted probability of diabetes mellitus at varying levels of walkability and NO_2_ among individuals remaining in their neighborhood for 5 or more years adjusted for baseline sociodemographic factors.

Walkability Quintiles (Q)	NO_2_ 0 ppb (SEM)	NO_2_ 5 ppb (SEM)	NO_2_ 10 ppb (SEM)	NO_2_ 20 ppb (SEM)	NO_2_ 30 ppb (SEM)	NO_2_ 40 ppb (SEM)
Q1 (lowest)	0.08 (0.002)	0.09 (0.002)	0.10 (0.001)	0.11 (0.001)	0.13 (0.003)	0.15 (0.005)
Q2	0.09 (0.002)	0.09 (0.002)	0.10 (0.001)	0.11 (0.001)	0.12 (0.003)	0.13 (0.004)
Q3	0.09 (0.002)	0.09 (0.002)	0.10 (0.001)	0.10 (0.001)	0.11 (0.002)	0.12 (0.003)
Q4	0.08 (0.003)	0.08 (0.002)	0.09 (0.002)	0.10 (0.001)	0.11 (0.002)	0.13 (0.004)
Q5 (Highest)	0.05 (0.003)	0.06 (0.002)	0.07 (0.002)	0.09 (0.001)	0.12 (0.003)	0.15 (0.006)

*Notes:* Covariates included in model: age, sex, ethnicity, immigration history, neighborhood median income. All covariates fixed at weighted average of levels for categorical covariates or mean value for continuous covariates. ppb: parts per billion. SEM: standard error of the mean.

Finally, we estimated models among a larger sample of individuals from the general population of adults aged 40–74 living in the same region at baseline, including those with and without a prior history of cardiovascular disease (N = 2,592,646) (Table 7). The estimated probabilities of hypertension and diabetes across levels of walkability and traffic-related air pollution, adjusted for sociodemographic variables, are found in Table 8, Table 9.

**Table 7:** Associations of walkability and traffic-related air pollution with hypertension and diabetes including individuals with prior cardiovascular disease or re-vascularization adjusted for baseline sociodemographic factors.

Variable	Hypertension		Diabetes	
	Independent Models OR (95% CI)	Joint Models OR (95% CI)	Independent Models OR (95% CI)	Joint Models OR (95% CI)
Walkability Quintile				
Q1 (low)	1.28 (1.25, 1.30)	1.34 (1.31, 1.37)	1.15 (1.12, 1.18)	1.24 (1.21, 1.28)
Q2	1.28 (1.25, 1.30)	1.33 (1.30, 1.35)	1.14 (1.11, 1.16)	1.21 (1.18, 1.24)
Q3	1.25 (1.23, 1.27)	1.29 (1.26, 1.31)	1.13 (1.11, 1.16)	1.19 (1.16, 1.22)
Q4	1.17 (1.14, 1.19)	1.19 (1.17, 1.21)	1.12 (1.09, 1.14)	1.16 (1.13, 1.18)
Q5 (high)	Ref	Ref	Ref	Ref
*p* for trend	<0.0001	<0.0001	<0.0001	<0.0001
Traffic-related air pollution				
NO_2_	1.02 (1.01, 1.03)	1.09 (1.07, 1.10)	1.11 (1.09, 1.12)	1.15 (1.13, 1.17)

*Notes* Covariates included in model: age, sex, ethnicity, immigration history, neighborhood median income). Association estimates for traffic-related air pollution are per 10-unit increase in NO_2_. Independent models include either walkability or traffic-related air pollution. Joint models include walkability and traffic-related air pollution simultaneously. OR: odds ratio, CI: confidence interval, Ref: reference category.

**Table 8:** Predicted probability of hypertension at varying levels of walkability and NO_2_ including individuals with prior cardiovascular disease or re-vascularization adjusted for baseline sociodemographic factors.

Walkability Quintiles (Q)	NO_2_ 0 ppb (SEM)	NO_2_ 5 ppb (SEM)	NO_2_ 10 ppb (SEM)	NO_2_ 20 ppb (SEM)	NO_2_ 30 ppb (SEM)	NO_2_ 40 ppb (SEM)
Q1 (lowest)	0.22 (0.003)	0.22 (0.002)	0.23 (0.002)	0.24 (0.002)	0.25 (0.003)	0.26 (0.006)
Q2	0.22 (0.004)	0.22 (0.003)	0.23 (0.002)	0.23 (0.002)	0.24 (0.003)	0.25 (0.005)
Q3	0.21 (0.003)	0.22 (0.002)	0.22 (0.002)	0.23 (0.001)	0.24 (0.003)	0.25 (0.004)
Q4	0.18 (0.004)	0.19 (0.003)	0.20 (0.002)	0.22 (0.001)	0.24 (0.003)	0.26 (0.005)
Q5 (Highest)	0.13 (0.005)	0.14 (0.004)	0.16 (0.003)	0.19 (0.001)	0.22 (0.003)	0.26 (0.007)

*Notes:* Covariates included in model: age, sex, ethnicity, immigration history, neighborhood median income. All covariates fixed at weighted average of levels for categorical covariates or mean value for continuous covariates. ppb: parts per billion. SEM: standard error of the mean.

**Table 9:** Predicted probability of diabetes mellitus at varying levels of walkability and NO_2_ including individuals with prior cardiovascular disease or re-vascularization adjusted for baseline sociodemographic factors.

Walkability Quintiles (Q)	NO_2_ 0 ppb (SEM)	NO_2_ 5 ppb (SEM)	NO_2_ 10 ppb (SEM)	NO_2_ 20 ppb (SEM)	NO_2_ 30 ppb (SEM)	NO_2_ 40 ppb (SEM)
Q1 (lowest)	0.09 (0.002)	0.10 (0.002)	0.10 (0.001)	0.12 (0.001)	0.14 (0.003)	0.16 (0.005)
Q2	0.10 (0.003)	0.10 (0.002)	0.10 (0.001)	0.11 (0.001)	0.12 (0.003)	0.13 (0.005)
Q3	0.09 (0.002)	0.10 (0.002)	0.10 (0.001)	0.11 (0.001)	0.12 (0.002)	0.13 (0.003)
Q4	0.08 (0.002)	0.09 (0.002)	0.10 (0.002)	0.11 (0.001)	0.13 (0.002)	0.14 (0.004)
Q5 (Highest)	0.06 (0.003)	0.06 (0.002)	0.07 (0.002)	0.09 (0.001)	0.12 (0.002)	0.16 (0.006)

*Notes:* Covariates included in model: age, sex, ethnicity, immigration history, neighborhood median income. All covariates fixed at weighted average of levels for categorical covariates or mean value for continuous covariates. ppb: parts per billion. SEM: standard error of the mean.

Table 10 includes the parameter estimates from logistic regression models assessing the interaction between traffic-related air pollution and neighborhood walkability on the odds of hypertension and diabetes. These models were fit among individuals free from cardiovascular disease at baseline.

**Table 10:** Parameter estimates from logistic regression model predicting hypertension and diabetes according to walkability and traffic-related air pollution.

Variable	Hypertension	Diabetes
	β (SE)	β (SE)
Intercept	−6.7383 (0.0439)	−6.5893 (0.0521)
Walkability Quintile		
Q1 (low)	0.6418 (0.0457)	0.5535 (0.0559)
Q2	0.6680 (0.0467)	0.6138 (0.0583)
Q3	0.6094 (0.0458)	0.6062 (0.0555)
Q4	0.4170 (0.0484)	0.4853 (0.0587)
Q5 (high)	Ref	Ref
Traffic-related air pollution (NO_2_, per 10 ppb)	0.2296 (0.0193)	0.3079 (0.0226)
Interaction Terms		
NO_2_ x Walkability Q1	−0.1683 (0.0224)	−0.1473 (0.0275)
NO_2_ x Walkability Q2	−0.1896 (0.0226)	−0.2040 (0.0287)
NO_2_ x Walkability Q3	−0.1700 (0.0216)	−0.2061 (0.0259)
NO_2_ x Walkability Q4	−0.1097 (0.0227)	−0.1558 (0.0274)
NO_2_ x Walkability Q5	Ref	Ref
Age (years)	0.0912 (0.0002)	0.0642 (0.0002)
Female	0.0098 (0.0035)	−0.2076 (0.0043)
Ethnicity		
Chinese	−0.2931 (0.0074)	−0.2223 (0.0110)
South Asian	0.1133 (0.0087)	0.6350 (0.0114)
General Population	Ref	Ref
Immigration History	−0.5662 (0.0110)	−0.3569 (0.0133)
5 or fewer years	−0.2164 (0.0086)	−0.0379 (0.0110)
Between 5 and 10 years	Ref	Ref
Over 10 years or Canadian-born		
Neighborhood Median		
Household Income Quintile		
Q1 (low)	0.3068 (0.0096)	0.5807 (0.013)
Q2	0.2989 (0.0083)	0.4759 (0.0121)
Q3	0.2340 (0.0082)	0.3818 (0.0123)
Q4	0.1588 (0.008)	0.2504 (0.0125)
Q5 (high)	Ref	Ref

NO_2_: nitrogen dioxide. Ref: reference category. SE: standard error. Q: quintile.

## 2 Experimental design, materials, and methods

### 2.1 Sample and data sources

Participants were drawn from the Cardiovascular Health in Ambulatory Care Research Team (CANHEART) cohort—a cohort of Canadian adults from Ontario, Canada assembled using administrative databases held at ICES in Toronto, Canada. The protocol for creation, individual databases used, and variables available have been described previously [9]. Selection criteria for sample used were described also elsewhere [5]. Briefly, individuals residing within one of 15 municipalities (Toronto & Greater Toronto Area, Hamilton, London, Ottawa) who were between 40 and 74 years of age were eligible for inclusion. Individuals who resided within a long-term care facility within the past 5 years were excluded. In total, data from 2,496,458 individuals was included.

Information on traffic-related air pollution was drawn from a national land use regression model designed to provide estimates of annual average outdoor NO_2_ concentration for locations across Canada [10]. Model predictors included satellite-derived NO_2_, land area for industrial uses within 2 km, total road length within 10 km, and summer rainfall. Estimates were generated for postal codes where individuals within our analytic sample resided. To additionally account for small-scale variation in NO_2_, deterministic gradients were used to adjust NO_2_ concentrations near major roads and highways, based on previously published estimates. The pollution estimates used were derived for 2006.

The information on neighborhood walkability used in the present analyses was derived from an index composed of 4 variables—population density (2006 Canadian Census), dwelling density (2006 Canadian Census), number of intersections with 3 or more intersecting roads/paths (2009 DMTI Spatial Inc.), and number of destinations (2009 DMTI Spatial Inc.) [11], [12], [13]. All variables were assessed using an 800 m network buffer.

Hypertension and diabetes were assessed using validated algorithms using diagnostic code information from individual's hospital admissions and outpatient physician billings, or fee codes for diabetes related programs. For hypertension, individuals who had one hypertension-related diagnostic code on a hospital admission or a hypertension-related diagnostic code on two outpatient physician service billings within a 2-year period before January 1 2008 were considered to have hypertension. Individuals were considered to have diabetes if they had records during a 2-year period before January 1 2008 indicating a diagnosis of diabetes during a hospital admission, two physician service billings, or a billing for a diabetes related program (i.e. insulin therapy support or diabetes management assessment). These algorithms were validated against clinical information, including laboratory testing information, clinical charts, measured blood pressures, and medication information. Validation of the hypertension algorithm found a sensitivity of 0.72 and specificity of 0.95 [14]. Validation of the diabetes detection algorithm found a sensitivity of 0.89 and 0.98 [15].

### 2.2 Analysis

Adjusted odds ratios were estimated using logistic regression models accounting for clustering at the neighborhood level (dissemination areas) using generalized estimating equations. Predicted probabilities were generated using logistic regression models including the main effects of walkability and traffic-related air pollution along with their multiplicative interaction term, in addition to covariates. The additional covariates included in the model are listed in the table notes and are further described by Howell and colleagues [5].
